# Underwater Bearing-Only and Bearing-Doppler Target Tracking Based on Square Root Unscented Kalman Filter

**DOI:** 10.3390/e21080740

**Published:** 2019-07-28

**Authors:** Xiaohua Li, Chenxu Zhao, Jing Yu, Wei Wei

**Affiliations:** 1 Shaanxi Key Laboratory for Network Computing and Security Technology, School of Computer Science and Engineering, Xi’an University of Technology, Xi’an 710048, China; 2 Science and Technology on Integrated Logistics Support Laboratory, National University of Defense Technology, Changsha 741200, China; 3 School of Marine Engineering Northwestern Polytechnical University, Xi’an 710072, China

**Keywords:** underwater, bearing-only, bearing-Doppler, square root unscented Kalman filter, observability, target tracking, Bayesian theory

## Abstract

Underwater target tracking system can be kept covert using the bearing-only or the bearing-Doppler measurements (passive measurements), which will reduce the risk of been detected. According to the characteristics of underwater target tracking, the square root unscented Kalman filter (SRUKF) algorithm, which is based on the Bayesian theory, was applied to the underwater bearing-only and bearing-Doppler non-maneuverable target tracking problem. Aiming at the shortcomings of the unscented Kalman filter (UKF), the SRUKF uses the QR decomposition and the Cholesky factor updating, in order to avoid that the process noise covariance matrix loses its positive definiteness during the target tracking period. The SRUKF uses sigma sampling to avoid the linearization of the nonlinear bearing-only and the bearing-Doppler measurements. To ensure the target state observability in underwater target tracking, the paper uses single maneuvering observer to track the single non-maneuverable target. The simulation results show that the SRUKF has better tracking performance than the extended Kalman filter (EKF) and the UKF in tracking accuracy and stability, and the computational complexity of the SRUKF algorithm is low.

## 1. Introduction

There are two major kinds of underwater target tracking system: a passive system [1] and an active system [2]. A passive system does not emit its own signal, and it acquires acoustic energy emitted by possible targets. An active system uses one or more transmitters and receivers; transmitter emits an acoustic signal and the receiver listens to echoes of this signal from a target [3]. Underwater passive target tracking, such as the bearing-only target tracking and the bearing-Doppler target tracking problem, are of great interest in a variety of underwater applications, for example in the military fields [1,2,3,4,5]. This is because that the sonar tracking system can be kept covert when using the bearing-only or the bearing-Doppler measurements (passive measurements), which will reduce the risk of been detected. Even so, there are some challenges that need to be solved for the underwater target tracking problem, such as the high degree of nonlinearity of the measurements and the target state range observability. 

The main issue that makes the underwater bearing-only and bearing-Doppler target tracking problem difficult is that the measurement processes are a high degree of nonlinearity [6]. In order to get the target’s states, the nonlinear Bayesian filtering algorithms are often used [7]. The most popular nonlinear and non-Gaussian Bayesian filter include the extended Kalman filter (EKF), the unscented Kalman filter (UKF) and the particle filter (PF). The EKF results only in first order accuracy of Taylor series expansion, which would make the tracker divergent if the target tracking system is seriously nonlinear and non-Gaussian [8]. The UKF uses a deterministic sampling method to capture targets’ posterior distribution of mean and covariance based on the unscented transform (UT) [9]. For the non-Gaussian systems, the approximations of the UKF are accurate to at least the second-order of Taylor series expansion [10]. For the PF, the mean and the standard covariance of the target are estimated by using the weighted particle sets at each tracking time [11,12]. However, the PF’s computational cost is large. The UKF has shown promise in the bearing-only and the bearing-Doppler target tracking problems. However, the process noise covariance matrix of the UKF algorithm often loses its positive definiteness because of the numerical instability. Consequently, the sigma points cannot be calculated correctly, which would make the tracking performance degraded. Also, the SRUKF can overcome those disadvantages using the QR decomposition, the Cholesky factor updating and the efficient least squares. 

The disadvantage of the Bayesian filters is that they may not work properly in the case of large initial estimation errors. In other words, the Bayesian filters may be sensitive to the targets’ initialization estimation. The modified pseudo measurement that was developed by A. Miller and B. Miller can exclude the bias in the bearing-only measurements [13,14,15]. We assume relatively good initialization in this paper.

The other challenging problem for the underwater bearing-only target tracking is that the target state may not be fully observable, i.e., the passive sensors do not have accurate information about the target range [16]. The target range observability issue can be solved by using a single maneuvering sensor or two or more stationary or maneuvering sensors if only the target does not move on the line of the multiple sensors. By introducing Doppler frequency measurement information, the passive target tracking system can be observable on the condition of without the observer’s maneuvering moving.

In this paper, we investigate the performance of the SRUKF algorithm for the single non-maneuvering target tracking based on bearing-only measurements and bearing-Doppler measurements for the cases of the single maneuvering observer. For the SRUKF algorithm, the covariance square root matrix is taken instead of covariance matrix in the UKF. In addition, the filtering divergence problem caused by non-positive error covariance matrix in the UKF is solved. In this paper, we consider the single non-maneuverable underwater target in the two-dimensional (2D) space.

The remaining of the paper is given as follows. Section 2 introduces the problem of the bearings-only and bearing-Doppler target tracking system model and measurement model. In Section 3, we give the implementation of the recursive Bayesian SRUKF algorithm. Simulation results and analysis are provided in Section 4 and conclusions are outlined in Section 5.

## 2. Problem Description

### 2.1. System Model

**Figure 1:**
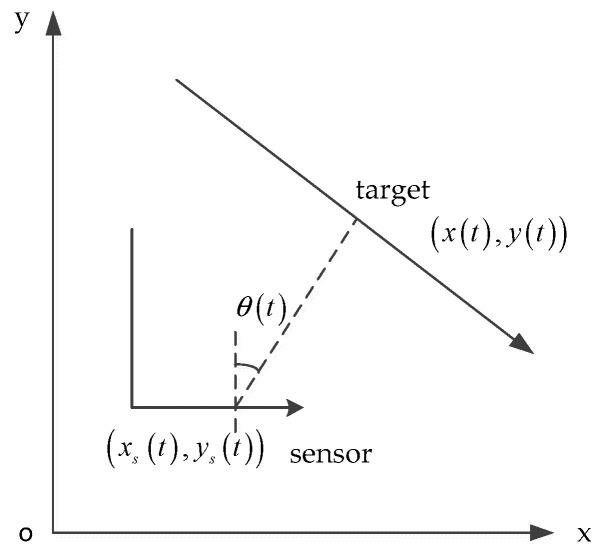
An overview of the target to observer (passive sensor) geometry.

In many cases, for an underwater target, the target’s movement is non-maneuvering. So, the nearly constant velocity (NCV) model [17,18] is appropriate in an underwater passive target tracking scenario. An overview of the target to single maneuvering observer (passive sensor) geometry for passive target tracking is shown in Figure 1.

In Figure 1, the target’s state vector is $x\left(t\right)={\left[x\left(t\right),y\left(t\right),\dot{x}\left(t\right),\dot{y}\left(t\right)\right]}^{T}$, where [x(t),y(t)] is the target’s location, and $\left[\dot{x}\left(t\right),\dot{y}\left(t\right)\right]$ is the target’s velocity. Similarly, the observer’s state is defined as $x_{s}^{}\left(t\right)={\left[x_{s}\left(t\right),y_{s}\left(t\right),{\dot{x}}_{s}\left(t\right),{\dot{y}}_{s}\left(t\right)\right]}^{T}$, where $\left[x_{s}\left(t\right),y_{s}\left(t\right)\right]$ is the observer’s location, and $\left[{\dot{x}}_{s}\left(t\right),{\dot{y}}_{s}\left(t\right)\right]$ is observer’s velocity.

For the NCV model, the target’s discrete-time state equation is:

(1) x(t)=F(t)x(t−1)+w(t),

where *t* is the sampling time, the w(t) is zero mean white Gaussian process noise with variance matrix Q(t), and F(t) is a deterministic transition matrix of the target. For the NCV model, we have:

(2) $$F\left(t\right)=\left[\begin{array}{cccc} 1 & 0 & \Delta t & 0\\ 0 & 1 & 0 & \Delta t\\ 0 & 0 & 1 & 0\\ 0 & 0 & 0 & 1 \end{array}\right],$$

(3) $$Q\left(t\right)=\left[\begin{array}{cccc} \frac{\Delta t^{3}}{3} & 0 & \frac{\Delta t^{2}}{2} & 0\\ 0 & \frac{\Delta t^{3}}{3} & 0 & \frac{\Delta t^{2}}{2}\\ \frac{\Delta t^{2}}{2} & 0 & \Delta t & 0\\ 0 & \frac{\Delta t^{2}}{2} & 0 & \Delta t \end{array}\right]\delta_{q}^{2},$$

where Δt is sampling interval, $\delta_{q}^{2}$ is system process noise intensity.

### 2.2. Bearing-Only Measurement Model

The bearing-only measurement’s discrete-time equation of the single maneuvering observer is given by:

(4) z(t)=h(t)+v(t),

where h(t) is bearing-only measurement function, v(t) is zero-mean independent Gaussian noise with variance R(t). 

The bearing-only measurement function h(t) is given by:

(5) $$h\left(t\right)=\arctan\left(\frac{x\left(t\right)-x_{s}\left(t\right)}{y\left(t\right)-y_{s}\left(t\right)}\right).$$

The target state of the bearing-only target tracking system may be unobservable. The bearing-Doppler target tracking system can overcome this problem. The Doppler frequency is determined by relative radial velocity between the target and the observer. Combine the bearing measurements, the Doppler frequency and relative radial velocity between the target and the observer, the system can get the distance from the observer to the target [19,20]. 

**Figure 2:**
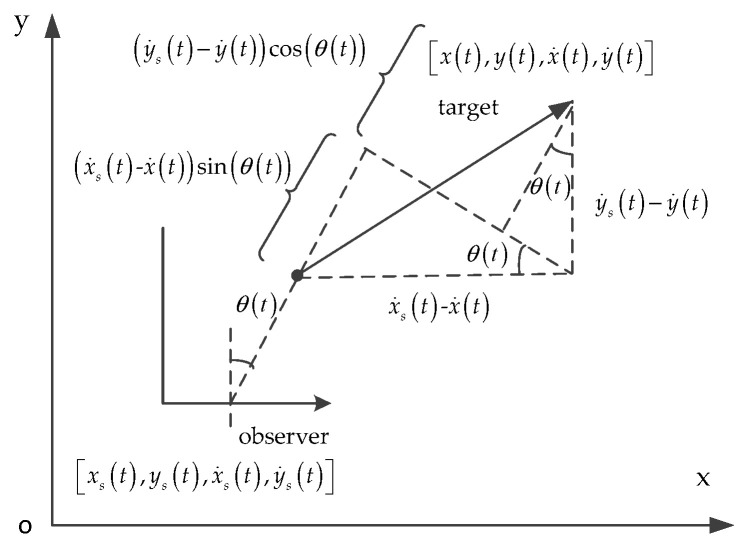
An overview of the Doppler-bearing tracking.

The overview of Doppler-bearing tracking is shown in Figure 2.

Take no account of the bearing-Doppler measurements noise, the Doppler frequency f(t) of the observer is given by:

(6) $$f\left(t\right)=f_{0}\left(1-\frac{v_{r}}{c}\right),$$

where c is the speed of sound in water, $v_{r}$ is the relative radial velocity between the target and observer, $f_{0}$ is the radiation frequency of the target, and:

(7) $$v_{r}=\left(\dot{x}\left(t\right)-{\dot{x}}_{s}\left(t\right)\right)\sin\theta \left(t\right)+\left(\dot{y}\left(t\right)-{\dot{y}}_{s}\left(t\right)\right)\cos\theta \left(t\right),$$

in which,

(8) $$\cos\theta \left(t\right)=\frac{y\left(t\right)-y_{s}\left(t\right)}{\sqrt{{\left(x\left(t\right)-x_{s}\left(t\right)\right)}^{2}+{\left(y\left(t\right)-y_{s}\left(t\right)\right)}^{2}}},$$

(9) $$\sin\theta \left(t\right)=\frac{x\left(t\right)-x_{s}\left(t\right)}{\sqrt{{\left(x\left(t\right)-x_{s}\left(t\right)\right)}^{2}+{\left(y\left(t\right)-y_{s}\left(t\right)\right)}^{2}}}.$$

Substituting the (7), (8) and (9) into (6), the Doppler frequency f(t) of the observer is given by:

(10) $$f\left(t\right)=\left[1-\frac{\left(\dot{x}\left(t\right)-{\dot{x}}_{s}\left(t\right)\right)\sin\theta \left(t\right)+\left(\dot{y}\left(t\right)-{\dot{y}}_{s}\left(t\right)\right)\cos\theta \left(t\right)}{c}\right]f_{0}.$$

Contrary to the bearing-only measurement, the measurement equation for the bearing-Doppler target tracking system is:

(11) $$z\left(t\right)=\left[\begin{array}{c} \arctan\left(\frac{x\left(t\right)-x_{s}\left(t\right)}{y\left(t\right)-y_{s}\left(t\right)}\right)\\ \left[1-\frac{\left(\dot{x}\left(t\right)-{\dot{x}}_{s}\left(t\right)\right)\sin\theta \left(t\right)+\left(\dot{y}\left(t\right)-{\dot{y}}_{s}\left(t\right)\right)\cos\theta \left(t\right)}{c}\right]f_{0} \end{array}\right]+v\left(t\right),$$

where v(t) is zero-mean independent Gaussian noise with variance matrix R(t), and

(12) $$R\left(t\right)=\left[\begin{array}{c} v_{\theta }\left(t\right)\\ v_{f}\left(t\right) \end{array}\right],$$

where $v_{\theta }\left(t\right)$ and $v_{f}\left(t\right)$ are bearing measurement noise and Doppler frequency noise covariance, respectively.

The bearing-Doppler measurement function h(t) is given by:

(13) $$\;h\left(t\right)=\left[\begin{array}{c} \arctan\left(\frac{x\left(t\right)-x_{s}\left(t\right)}{y\left(t\right)-y_{s}\left(t\right)}\right)\\ \left[1-\frac{\left(\dot{x}\left(t\right)-{\dot{x}}_{s}\left(t\right)\right)\sin\theta \left(t\right)+\left(\dot{y}\left(t\right)-{\dot{y}}_{s}\left(t\right)\right)\cos\theta \left(t\right)}{c}\right]f_{0} \end{array}\right].$$

## 3. Bayesian Filtering

Bayesian filtering is based on the Bayesian principle [21,22,23]. For the Bayesian filtering, the target state is regarded as a random variable, which is an estimation of the prior information of the observation data (measurements) of the target and environment noise. Bayesian filtering converts the target state and measurements from the state space to probability distribution. The goal of Bayesian filtering is to estimate the state of a nonlinear dynamic process conditioned on the measurements. 

The principle of Bayesian filtering is to predict the system state by using the prior probability density function of the system model firstly, and then to update the posterior probability density function with the latest observation information (measurements) [24,25]. For this paper, the measurements are bearings-only and bearing-Doppler.

The Bayesian filtering includes two steps: state prediction and state updating. 

Assuming the target states satisfy the hidden Markov process, the target state model and measurement model are the same with the system model and measurement model in the Section 2, i.e., the target state model is Equation (1), and the measurement models are Equations (4) and (11). The measurement at the t time step is dependent upon the current target state. Then, the procedures of the Bayesian filtering are given as follows:

State prediction:

(14) p(x(t)|z(0:t−1))=∫p(x(t)|x(t−1))p(x(t−1)|z(0:t−1))dx(t−1).

State updating:

(15) $$p\left(x\left(t\right)|z\left(0:t\right)\right)=\frac{p\left(z\left(t\right)|x\left(t\right)\right)p\left(x\left(t\right)|z\left(0:t-1\right)\right)}{p\left(z\left(t\right)|z\left(0:t-1\right)\right)},$$

where

(16) p(z(t)|z(0:t−1))=∫p(z(t)|x(t))p(x(t)|z(0:t−1))dx(t),

where p(⋅) is the probability density function (PDF).

These two recursive steps of Equations (13) and (14) constitute the recursive Bayesian filtering. Equation (13) represents the target state propagation, while Equation (14) is the measurement update. For the linear and Gaussian target tracking system, the Kalman filter is the optimization method. For the nonlinear and non-Gaussian target tracking system, the EKF, the UKF, the PF, and their improved algorithms are often used. 

## 4. Square Root Unscented Kalman Filter

The square root unscented Kalman filter algorithm is based on the unscented Kalman filter. In order to improve the efficiency and the stability of the UKF algorithm, the SRUKF makes use of three powerful linear algebra methods: QR decomposition, Cholesky factor updating and efficient least squares. 

For the SRUKF, the covariance square root matrix is taken instead of the covariance matrix in the UKF. In addition, for the SRUKF, the filtering divergence problem caused by non-positive error covariance matrix in the UKF is solved.

### 4.1. Unscented Kalman Filter

Contrary to the EKF, the UKF uses a deterministic sampling method to capture targets’ posterior distribution of mean and covariance based on the unscented transform. For non-Gaussian inputs, the UKF’s approximations are accurate to at least the second-order of Taylor series expansion [26,27,28]. For Gaussian inputs, the UKF can result in the third order of Taylor series expansion for all nonlinearities. 

**Figure 3:**
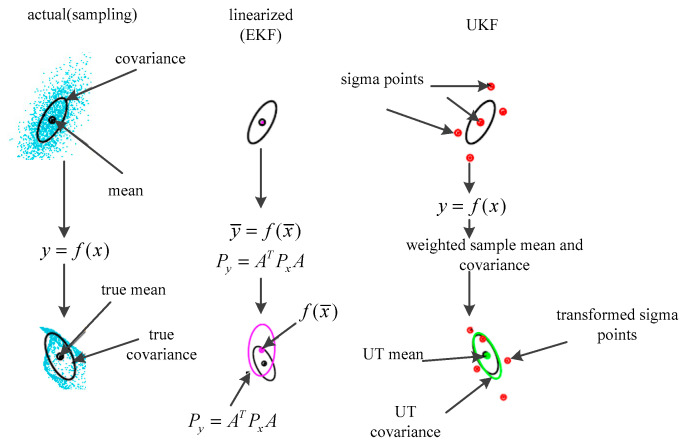
Example of mean and covariance propagation: actual, first-order linearization of EKF, sampling method of the UKF.

An example of mean and covariance propagation of the actual, first-order linearization of EKF and sampling method of the UKF is given in Figure 3 [9]. In Figure 3, the left parts represent the true mean and covariance propagation using the Monte Carlo sampling method; the center parts represent the propagation results using the non-linearization approach of the EKF; the right parts represent the results using the new sampling approach of the UKF. The implementation of the UKF is shown below [29]:

Assume the target state vector is *n*-dimension and measurement vector is *m*-dimension. Then, the number of the sigma points in UKF is 4n+2m+1. Extending the dimension of the target state vector and its covariance matrix, the initialization of UKF is as follows:

(17) $${\hat{x}}^{a}\left(0\right)=E\left(x^{a}\left(0\right)\right)={\left[\begin{array}{ccc} {\hat{x}}^{T}\left(0\right) & 0 & 0 \end{array}\right]}^{T},$$

(18) $${\hat{p}}^{a}\left(0\right)=E\left[\left(x^{a}\left(0\right)-{\hat{x}}^{a}\left(0\right)\right){\left(x^{a}\left(0\right)-{\hat{x}}^{a}\left(0\right)\right)}^{T}\right]={\left[\begin{array}{ccc} \hat{p}\left(0\right) & 0 & 0\\ 0 & Q & 0\\ 0 & 0 & R \end{array}\right]}^{T},$$

where ${\hat{x}}^{a}\left(0\right)$ and ${\hat{p}}^{a}\left(0\right)$ denote the mean and covariance of the augmented state vector, respectively. Then, the dimension of augmented state is L=2n+m. 

The UKF algorithm is presented below. 

Calculate sigma points:

(19) $$\chi \left(t-1\right)={\left[\begin{array}{ccc} {\hat{x}}^{a}\left(t-1\right) & {\hat{x}}^{a}\left(t-1\right)+\sqrt{\left(L+ƛ\right){\hat{P}}^{a}\left(t-1\right)} & {\hat{x}}^{a}\left(t-1\right)-\sqrt{\left(L+ƛ\right){\hat{P}}^{a}\left(t-1\right)} \end{array}\right]}^{T}.$$

Time update:

(20) χ(t|t−1)=F(χ(t−1)),

(21) $$\hat{x}\left(t|t-1\right)=\sum_{i=0}^{2L}W_{i}^{m}\chi_{i}^{}\left(t|t-1\right),$$

(22) $$\hat{P}\left(t|t-1\right)=\sum_{i=0}^{2L}W_{i}^{c}\left(\chi_{i}^{}\left(t|t-1\right)-\hat{x}\left(t|t-1\right)\right){\left(\chi_{i}^{}\left(t|t-1\right)-\hat{x}\left(t|t-1\right)\right)}^{T},$$

(23) Z(t|t−1)=h(χ(t|t−1)),

(24) $$\hat{z}\left(t|t-1\right)=\sum_{i=0}^{2L}W_{i}^{m}Z_{i}^{}\left(t|t-1\right).$$

Measurement update:

(25) $${\hat{P}}_{zz}^{}\left(t\right)=\sum_{i=0}^{2L}W_{i}^{c}\left(Z_{i}^{}\left(t|t-1\right)-\hat{z}\left(t|t-1\right)\right){\left(Z_{i}^{}\left(t|t-1\right)-\hat{z}\left(t|t-1\right)\right)}^{T},$$

(26) $${\hat{P}}_{xz}^{}\left(t\right)=\sum_{i=0}^{2L}W_{i}^{c}\left(\chi_{i}^{}\left(t|t-1\right)-\hat{x}\left(t|t-1\right)\right){\left(Z_{i}^{}\left(t|t-1\right)-\hat{z}\left(t|t-1\right)\right)}^{T},$$

(27) $$G\left(t\right)={\hat{P}}_{xz}^{}\left(t\right){\hat{P}}_{zz}^{}{\left(t\right)}^{-1},$$

(28) $$\hat{x}\left(t\right)=\hat{x}\left(t|t-1\right)+G\left(t\right)\left(z\left(t\right)-\hat{z}\left(t|t-1\right)\right),$$

(29) $$\hat{P}\left(t\right)=\hat{P}\left(t|t-1\right)-G\left(t\right){\hat{P}}_{zz}^{}\left(t\right)G{\left(t\right)}^{T},$$

where the weights $W_{i}^{m}$ and $W_{i}^{c}$ are defined as

(30) $$\left\{ \begin{array}{l} W_{0}^{m}=ƛ/\left(L+ƛ\right)\\ W_{0}^{c}=ƛ/\left(L+ƛ\right)+\left(1-\alpha^{2}+\beta \right)\\ W_{0}^{c}=W_{0}^{m}=1/\left[2\left(L+ƛ\right)\right] \end{array}\right.,$$

where $ƛ=\alpha^{2}\left(L+\kappa \right)-L$, and α, β and κ are scaling parameters.

### 4.2. Square Root Unscented Kalman Filter

In order to improve the efficiency and the stability of the UKF, the SRUKF makes use of three powerful linear algebra methods: QR decomposition, the Cholesky factor updating and efficient least squares [30,31], which are briefly given below:

QR decomposition:

If a positive definite matrix $A\in {ℜ}^{L\times N}$, the QR decomposition of the matrix A is given by $A^{T}=QR$, in which $Q\in {ℜ}^{N\times N}$ is orthogonal, and $R\in {ℜ}^{N\times N}$ is a upper triangular and N≥L. Define the upper triangular part of R as $\bar{R}$, then $\bar{R}$ is the transpose of the Cholesky factor of the positive definite matrix $P=AA^{T}$, i.e., $\bar{R}=S^{T}$, such that ${\bar{R}}^{T}\bar{R}=AA^{T}$. 

This paper uses qr{⋅} to denote the QR decomposition of a matrix, where $\bar{R}$ is the only return value. 

Cholesky factor update:

Denote the Cholesky factor of updating $P\pm \sqrt{\nu }uu^{T}$ as S=cholupdate{S,u,±ν}, in which S is the original Cholesky factor of $P=AA^{T}$. 

If u is a matrix and not a vector, then the result is l consecutive updates of the Cholesky factor using the l columns of u. 

Efficient least square:

In order to get the solution of the equation $\left(AA^{T}\right)x=A^{T}b$, one can use the solution of the efficient least squares problem Ax=b. The least squares problem can be solved efficiently using a QR decomposition.

The SRUKF propagates and updates the target’s covariance matrix using the Cholesky factor. The main advantage of the SRUKF is that it only saves the square root of the covariance matrix, which would reduce the computational cost. In addition, the SRUKF can ensure tracking stability, as it is meaningful only if the covariance matrix is a nonnegative definite matrix—which the SRUKF can insure this [32].

The implementation of the SRUKF is given below:

1. Initialization:
  (31) $${\hat{x}}_{0}=E\left[x_{0}\right],S_{0}=\mathrm{chol}\left\{E\left[\left(x_{0}-{\hat{x}}_{0}\right){\left(x_{0}-{\hat{x}}_{0}\right)}^{T}\right]\right\},$$
  where chol{⋅} denotes the Cholesky factorization.
2. Calculate the sigma points:
  (32) $$\chi \left(t-1\right)={\left[\begin{array}{ccc} \hat{x}\left(t-1\right) & \hat{x}\left(t-1\right)+\sqrt{\left(L+ƛ\right)}S\left(t-1\right) & {\hat{x}}^{a}\left(t-1\right)-\sqrt{\left(L+ƛ\right)}S\left(t-1\right) \end{array}\right]}^{T},$$
  where L is the dimension of augmented target state, $ƛ=\alpha^{2}\left(L+\kappa \right)-L$, and α, β and κ are scaling parameters, which are the same with the UKF.
3. Time update:
  (33) χ(t|t−1)=F(χ(t−1))
  (34) $$\hat{x}\left(t|t-1\right)=\sum_{i=0}^{2L}W_{i}^{m}\chi_{i}^{}\left(t|t-1\right)$$
  (35) $$S\left(t|t-1\right)=qr\left\{\left[\sqrt{W_{1}^{c}}\left(\chi_{1:2L}\left(t|t-1\right)-\hat{x}\left(t|t-1\right)\right)\sqrt{Q}\right]\right\}$$
  (36) $$S\left(t|t-1\right)=\mathrm{cholupdate}\left\{S\left(t|t-1\right),\chi_{0}\left(t|t-1\right)-\hat{x}\left(t|t-1\right),W_{0}^{c}\right\}$$
  (37) Z(t|t−1)=h(χ(t|t−1))
  (38) $$\hat{z}\left(t|t-1\right)=\sum_{i=0}^{2L}W_{i}^{m}Z_{i}^{}\left(t|t-1\right)$$
  where Q is process noise variance matrix, and the weights $W_{i}^{m}$ and $W_{i}^{c}$ are same with the UKF algorithm above. The qr{⋅} denotes the QR decomposition of a matrix, and the cholupdate{S,χ,W} returns the Cholesky factor.
4. Measurements update:
  (39) $$S_{z}\left(t|t\right)=\mathrm{qr}\left\{\left[\sqrt{W_{1}^{c}}\left(Z_{1:2L}\left(t|t-1\right)-\hat{z}\left(t|t-1\right)\right)\sqrt{R}\right]\right\}$$
  (40) $$S_{z}\left(t|t\right)=\mathrm{cholupdate}\left\{S_{z}\left(t|t\right),Z_{0}\left(t|t-1\right)-\hat{z}\left(t|t-1\right),W_{0}^{c}\right\}$$
  (41) $${\hat{P}}_{xz}^{}\left(t\right)=\sum_{i=0}^{2L}W_{i}^{c}\left(\chi_{i}^{}\left(t|t-1\right)-\hat{x}\left(t|t-1\right)\right){\left(Z_{i}^{}\left(t|t-1\right)-\hat{z}\left(t|t-1\right)\right)}^{T}$$
  (42) $$G\left(t\right)=\left({\hat{P}}_{xz}^{}\left(t\right)/S_{z}^{T}\left(t|t\right)\right)/S_{z}\left(t|t\right)$$
  (43) $$\hat{x}\left(t\right)=\hat{x}\left(t|t-1\right)+G\left(t\right)\left(z\left(t\right)-\hat{z}\left(t|t-1\right)\right)$$
  (44) $$S\left(t|t\right)=\mathrm{cholupdate}\left\{S\left(t|t-1\right),G\left(t\right)S_{z}\left(t|t\right),-1\right\}$$
  where R is measurement noise variance matrix.

The tracking accuracy and the filter stability of the SRUKF method are better than the UKF. In addition, the SRUKF only preserves the square root of the target’s covariance matrix, not the whole covariance matrix, as this would reduce the computational cost. The other advantage of the SRUKF algorithm is that it can make the target covariance matrix to be a nonnegative definite matrix.

## 5. Simulations

This section considers the two tracking scenarios: the single maneuvering observer for the bearing-only scenario and the single maneuvering observer for the bearing-Doppler scenario. Both scenarios treat the same single nearly constant velocity moving target. 

The target’s original bearing is $30^{\circ }$, the original moving course is $140^{\circ }$ and the moving speed is 40 Kn. The target’s initial location is (900, 1700) m and the velocity is (25, −30) m/s. The total tracking time is 200 s with the sampling interval Δt=1s. The Monte Carlo runs is 100. The single maneuvering observer’s initial location is (0, −1000) m and the initial velocity is (0, 6) m/s. In the middle time scan of the experiment, the observer is turning with velocity (6, 6) m/s. The sound speed of underwater is 1500 m/s, and the target’s radiant frequency is $f_{0}=385\;\mathrm{Hz}$. The system process noise and bearing-only and bearing-Doppler measurement noise satisfy the white Gaussian noise distribution. The system process noise intensity is $\delta_{q}^{2}=0.3\;m$, the bearing noise covariance is $v_{\theta }\left(t\right)=7^{\circ }$, and the Doppler noise covariance is $v_{f}\left(t\right)=15\;\mathrm{Hz}$. 

The root-mean square error (RMSE) of position is defined as:

(45) $$RMSE\left(t\right)=\sqrt{\frac{1}{M}\sum_{m=1}^{M}\left[{\left(x_{m}\left(t\right)-{\tilde{x}}_{m}\left(t\right)\right)}^{2}+{\left(y_{m}\left(t\right)-{\tilde{y}}_{m}\left(t\right)\right)}^{2}\right]}$$

where M is the total runs of Monte Carlo, $x_{m}\left(t\right)$ and $x_{y}\left(t\right)$ are the true state of target, and ${\tilde{x}}_{m}\left(t\right)$ and ${\tilde{y}}_{m}\left(t\right)$ are the estimated state.

### 5.1. Case of Bearing-only Measurement

**Figure 4:**
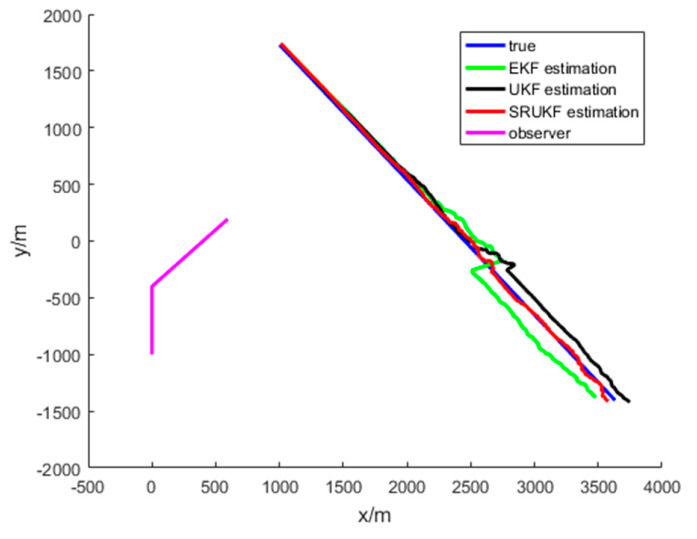
The bearing-only tracking scenario: target’s true trajectory and the EKF (green), the UKF (black) and the SRUKF (red) estimated trajectory.

**Figure 5:**
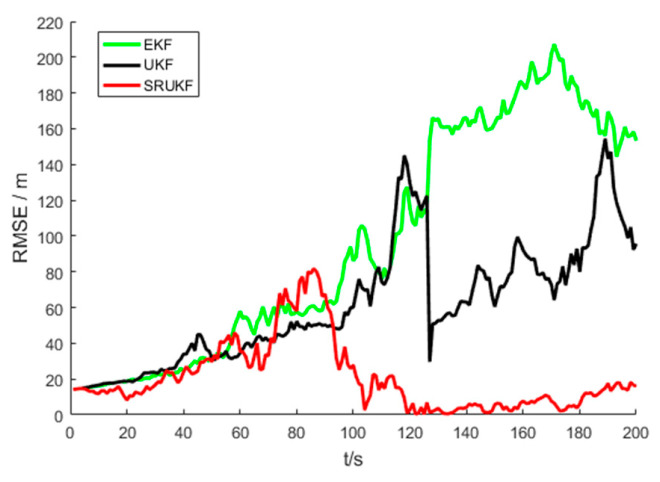
The RMSE of position for bearing-only tracking scenario, EKF (**green**), UKF (**black**) and SRUKF (**red**) estimation.

**Figure 6:**
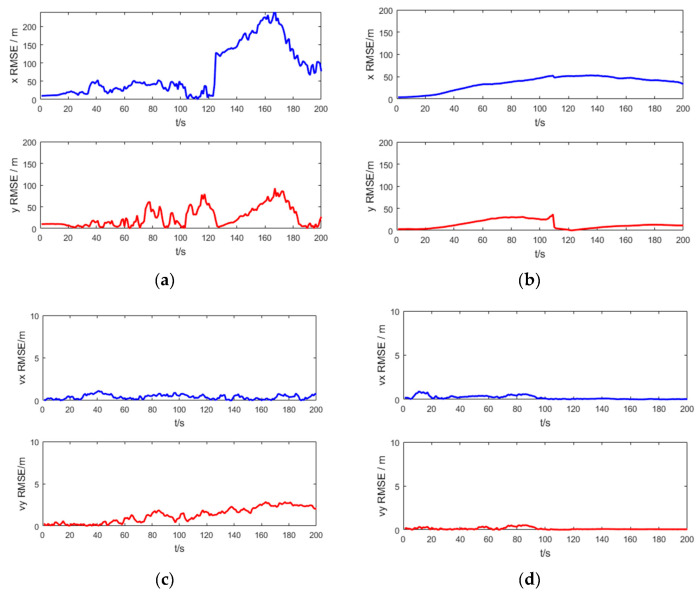
The RMSE of target position and velocity for the bearing-only target tracking scenario: (**a**) position RMSE of the UKF; (**b**) position RMSE of the SRUKF, (**c**) velocity RMSE of the UKF; (**d**) velocity RMSE of the SRUKF.

**Figure 7:**
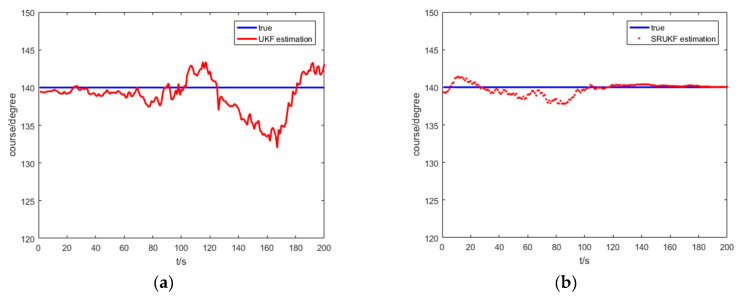
The true course and the UKF and the SRUKF estimated course for bearing-only target tracking scenario: (**a**) true course and the UKF estimated course; (**b**) true course and the SRUKF estimated course.

**Figure 8:**
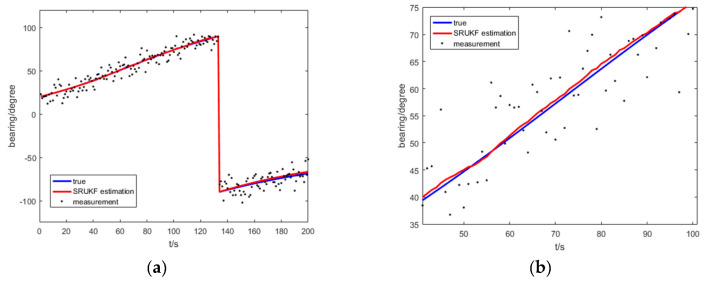
The true bearing, the measurement and SRUKF estimated bearing for bearing-only tracking scenario: (**a**) the total scan; (**b**) local enlarged figure of (**a**).

The simulation results for the bearing-only target tracking scenario are shown in Figure 4, Figure 5, Figure 6, Figure 7 and Figure 8. Figure 4 gives the scenario of true target trajectory and the estimated target trajectory. As seen in Figure 4, the SRUKF can track the target efficiently in the whole tracking period, and in the second half of the tracking period, the EKF and the UKF algorithm tend to divergent. The tracking performance of the SRUKF is superior to the EKF and UKF, and the UKF’s tracking accuracy is better than the EKF.

The Figure 5 shows the performance of bearing-only target tracking system in the sense of root mean square error (RMSE) of the target position versus time scans for the EKF, the UKF and the SRUKF. It can be clearly seen that the RMSE of the SRUKF is the smallest. Along with the tracking time going on, the RMSE of the EKF and the UKF is increased, and the tracking performance of the EKF and the UKF are divergent. This also shows that the tracking accuracy of the SRUKF is better than the EKF and the UKF. 

Figure 6 shows the RMSE of the target position and velocity of the UKF and the SRUKF for the bearing-only tracking scenario, in which the x RMSE and y RMSE denote the RMSE of the x axis and y axis, respectively, and the vx RMSE and xy RMSE denote the RMSE of the velocity in x axis and y axis, respectively. As shown in Figure 6, in the second half of the tracking period, the x RMSE, y RMSE, vx RMSE and vy RMSE of the UKF is larger than the SRUKF, which is consistent with the Figure 6. 

Figure 7 shows the true course, and the UKF and the SRUKF estimated course of the target for the bearing-only target tracking system. As shown in Figure 7, the SRUKF estimated course is closer to the target’s true course than the UKF. This also exhibits that the error performance of the SRUKF is superior to the UKF. 

Figure 8 shows the true bearing, the SRUKF estimated bearing and measurement of bearing for the target for the bearing-only target tracking scenario. To take a close look, Figure 8b enlarges the local image of the Figure 8a. As is shown in Figure 8, the SRUKF estimated bearings are close to the true bearings.

### 5.2. Case of Bearing-Doppler Measurement

**Figure 9:**
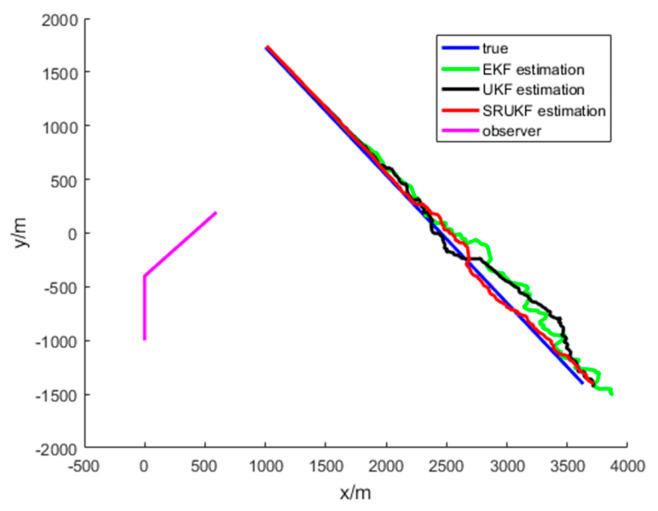
The bearing-Doppler tracking scenario: target’s true trajectory and EKF (green), UKF (black) and SRUKF (red) estimated trajectory.

**Figure 10:**
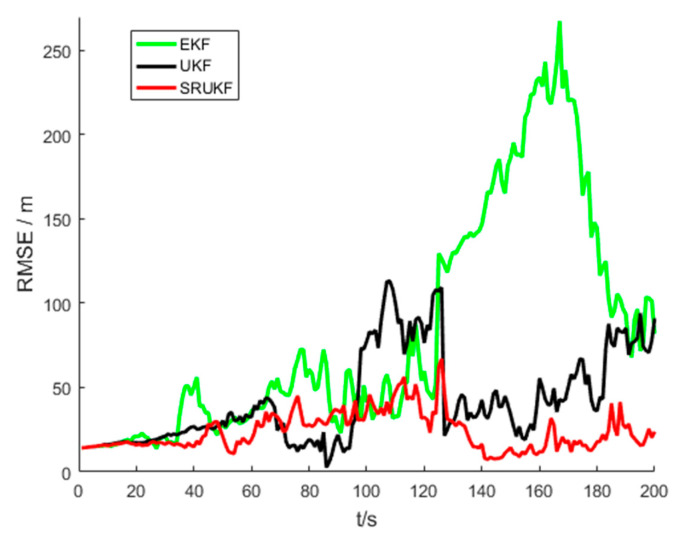
The RMSE of position for bearing-Doppler tracking scenario, EKF (**green**), UKF (**black**) and SRUKF (**red**) estimation

**Figure 11:**
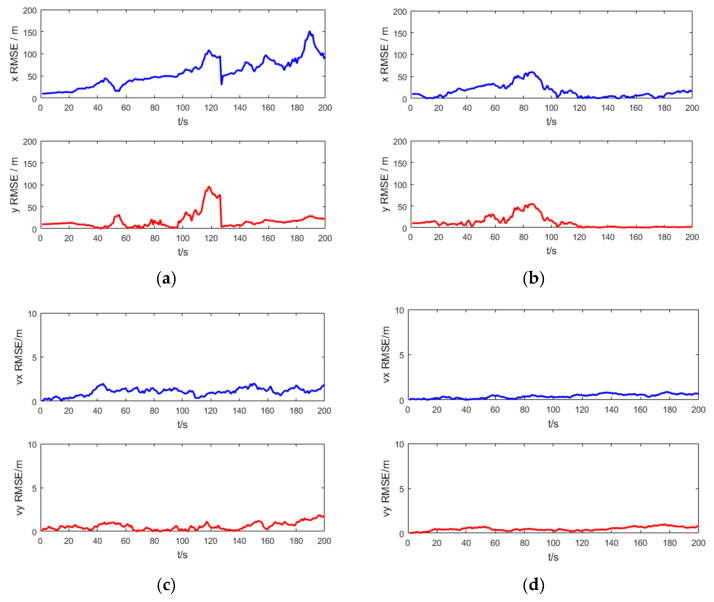
The RMSE of position and velocity for bearing-Doppler tracking scenario: (**a**) position RMSE of the UKF; (**b**) position RMSE of the SRUKF, (**c**) velocity RMSE of the UKF; (**d**) velocity RMSE of the SRUKF.

**Figure 12:**
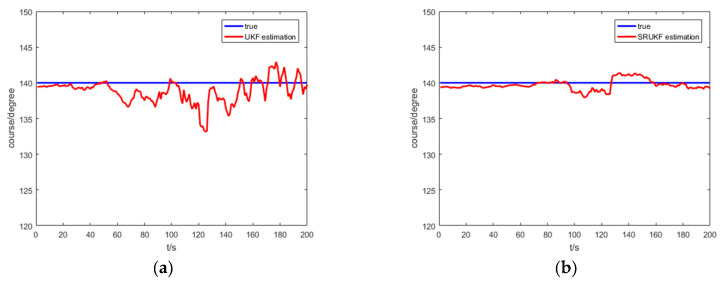
The true course and the UKF and the SRUKF estimated course for bearing-Doppler target tracking scenario: (**a**) true course and the UKF estimated course; (**b**) true course and the SRUKF estimated course.

**Figure 13:**
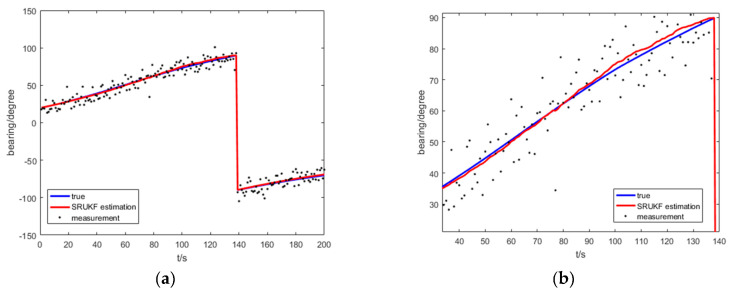
The true bearing, the measurement and SRUKF estimated bearing for bearing-Doppler tracking scenario: (**a**) the total scan; (**b**) local enlarged figure of (**a**).

**Figure 14:**
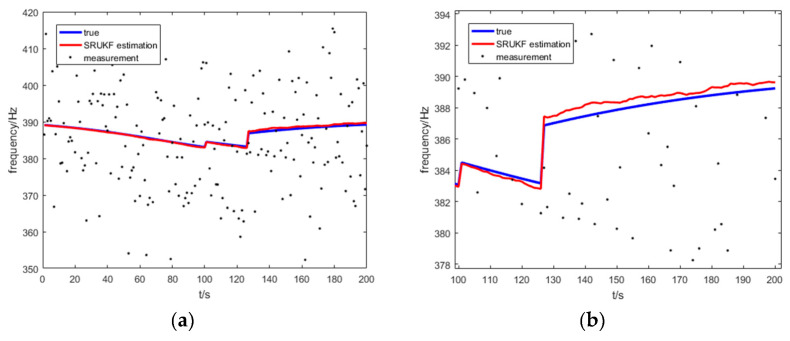
The true frequency, the measurement and the SRUKF estimated frequency for the bearing-Doppler target tracking scenario: (**a**) the total tracking scan; (**b**) local enlarged figure of (**a**).

The simulation results for the bearing-Doppler target tracking scenario are shown in Figure 9, Figure 10, Figure 11, Figure 12, Figure 13 and Figure 14. Figure 9 gives the scenario of the target’s true trajectory and the EKF, the UKF and the SRUKF estimated trajectory. As seen in Figure 9—in the second half of the tracking period—the EKF and the UKF tend to divergent. The SRUKF estimated trajectory is close to the true trajectory, which means that the SRUKF algorithm can track the target efficiently in the whole tracking period. It is easy to see that the tracking performance of the SRUKF is superior to the EKF and the UKF, and the UKF’s tracking accuracy is better than the EKF. In addition, the computational cost of the SRUKF algorithm is small.

Figure 10 shows the performance of bearing-Doppler target tracking system in the sense of RMSE of the target position versus time scans for the EKF, the UKF and the SRUKF. Similar to the bearing-only target tracking scenario, it can be clearly seen that the position RMSE of the SRUKF algorithm is the smallest, and that the position RMSE of the UKF is smaller than the EKF algorithm. Along with the tracking time going on, the position RMSE of the EKF is increased, and the tracking for the EKF is divergent. This also shows that the tracking accuracy of the SRUKF is better than the EKF and the UKF. 

As seen in Figure 5 and Figure 10, the bearing-only target tracking system exhibits a larger RMSE in position than the bearing-Doppler target tracking system. This is because the bearing-Doppler system has one more measurement information, i.e., Doppler, than the bearing-only tracking system. Also, the SRUKF algorithm has small RMSE in position for the both systems. 

Figure 11 shows the RMSE of the target position and velocity of the UKF and the SRUKF for the bearing-Doppler target tracking scenario. As shown in Figure 11, in the second half of the tracking period, the x RMSE, y RMSE, vx RMSE and vy RMSE of the UKF is larger than the SRUKF, which is similar with the Figure 6. Also, the RMSE in velocity are small for both the bearing-only tracking system and the bearing-Doppler tracking systems. Good tracking performance is maintained over the simulation period for the SRUKF algorithm. 

Figure 12 shows the true course; the UKF and the SRUKF estimated course of the target for the bearing-Doppler target tracking system. As shown in Figure 12, the SRUKF estimated course is close to the target’s true course—except for some sampling scan. Also, the differences between the UKF estimated course and the true course is large. This is also exhibits that the accuracy performance of the SRUKF is superior to the UKF.

Figure 13 shows the true bearing of the SRUKF estimated bearing and measurement of bearing of the target for the bearing-Doppler target tracking scenario. Seen more clearly, Figure 13b enlarges the local image of the Figure 13a. As is shown in Figure 13, the SRUKF estimated bearings are close to the true bearings.

Figure 14 shows the true frequency, the SRUKF estimated frequency and measurement of frequency of the target for the bearing-Doppler target tracking scenario. Seen more clearly, Figure 14b enlarges the local image of the Figure 14a. As is shown in Figure 14, the differences between the SRUKF estimated frequencies and the true bearings is small. This is because the process noise covariance matrix often loses its positive definiteness because of the numerical instability during the tracking period. Consequently, the sigma points cannot be correctly calculated. Also, the SRUKF can overcome this disadvantage using the QR decomposition and the Cholesky factor updating. 

## 6. Conclusions

The main advantage of the underwater target tracking using the bearing-only or the bearing-Doppler measurements (passive measurements) is that the sonar can be kept covert, which will reduce the risk of being detected. According to the characteristics of underwater target tracking, the Bayesian filtering algorithm SRUKF was applied to the underwater bearing-only and bearing-Doppler non-maneuverable target tracking problem. To ensure the range observability in passive underwater target tracking, we addressed the bearings-only and bearing-Doppler target tracking problem with single maneuvering observer to track the single non-maneuverable target. The tracking accuracy and the filter stability of the SRUKF method are better than the UKF method. In addition, the SRUKF only preserved the square root of the covariance matrix (not the whole covariance matrix), which reduced the computational cost. The other advantage of the SRUKF algorithm is that it can make the covariance matrix to be a nonnegative definite matrix. The simulation results show that the SRUKF has better tracking performance than the EKF and the UKF in tracking accuracy and stability, and that the computational complexity of the SRUKF algorithm is low.
